# Tranexamic acid does not have a dose-dependent effect on postoperative delirium after cardiac surgery – a retrospective cohort study

**DOI:** 10.1051/ject/2025025

**Published:** 2025-12-17

**Authors:** Rachel Wong, Scott Minns, Florian Falter

**Affiliations:** 1 Royal Papworth Hospital Cambridge Biomedical Campus Cambridge CB2 0AY UK; 2 University Hospitals Coventry and Warwickshire Coventry DV2 2DX UK

**Keywords:** Cardiac surgery, Cardiopulmonary bypass, Postoperative delirium, Postoperative neurocognitive decline, Tranexamic acid, Outcome

## Abstract

*Background*: Postoperative delirium, regularly seen after cardiac surgery, is challenging. It has significant implications for healthcare resources and significant implications for individual patients and their families. Although the exact mechanisms are not understood, there is emerging evidence that blood-brain-barrier disruption and neuroinflammation may play a role in developing postoperative delirium. Tranexamic acid, frequently used in cardiac surgery for its transfusion-sparing effect, has recently been shown to ameliorate neuroinflammation and stabilise the blood-brain barrier. This study investigates if there is a dose-dependent effect of tranexamic acid on developing postoperative delirium after cardiac surgery on cardiopulmonary bypass. *Methods*: 5525 patients were included in this retrospective, observational database study. Patients were divided into three groups, depending on the dose of tranexamic acid they had received before heparinisation (Group A (*n* = 1780) up to 22 mg/kg, Group B (*n* = 2130) 22.01 – 27 mg/kg, and Group C (*n* = 1615) 27.01 mg/kg or more). All three doses are clinically relevant and seen regularly. The presence of postoperative delirium was defined by at least one “CAM-ICU positive” entry in the patient’s medical record. *Results*: There was no statistically significant difference between the three groups in the incidence of postoperative delirium. The percentage of CAM-ICU-positive patients in each group was in keeping with the overall cohort (Overall = 18%, Group A = 18%, Group B = 17%, Group C = 20%, p = 0.25). *Conclusion*: The results do not support the theory that tranexamic acid given in the higher clinically acceptable dose range decreases the incidence of postoperative delirium after cardiac surgery.

## Introduction

Postoperative delirium (PD) presents a significant challenge, with increased morbidity and frailty, functional decline, length of intensive care and hospital stay, need for long-term care, and overall quality of life and mortality [1, 2]. PD is particularly prevalent after cardiac surgery, with some studies reporting an incidence of over 50% [3]. Identifying and managing modifiable risk factors have long been subjects of clinical and academic interest to optimise outcomes after cardiac surgery [4].

**Figure 1:**
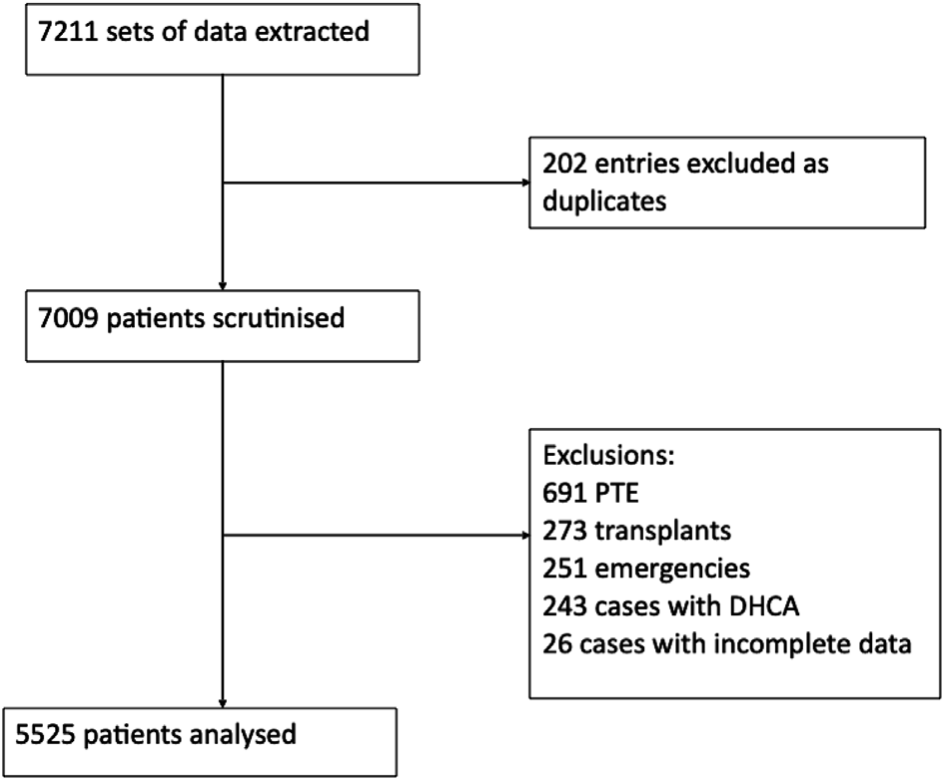
STROBE diagram. PTE = pulmonary thrombendarterectomy, DHCA = deep hypothermic circulatory arrest.

**Table 1:** Summary of patient demographic. TXA = Tranexamic Acid, CABG = coronary artery bypass grafting, CPB = cardiopulmonary bypass, AOX = aortic cross clamp.

	All patients (*n* = 5521)	CAM-ICU negative (*n* = 4519, 82%)	CAM-ICU positive (*n* = 1002, 18%)	*p-*value
TXA (mg/kg)	24.1 [24.1–24.39]	24.1 [20.8–27.8]	24.7 [21.1–28.6]	0.07
Age	71 [71–71]	70 [62–76]	73 [66–78]	<0.001
Sex				
Male	4013	3319 (83%)	694 (17%)	
Female	1508	1200 (80%)	308 (20%)	
Left ventricular function				
Good	3691	3099 (84%)	592 (16%)	
Moderate	1517	1199 (79%)	318 (21%)	
Poor	217	155 (71%)	62 (29%)	
Very poor	96	66 (69%)	30 (31%)	
Hypertension				
Yes	2998	2422 (81%)	576 (19%)	
No	2523	2097 (83%)	426 (17%)	
Diabetes mellitus				
Yes	1618	1270 (78%)	348 (22%)	
No	3903	3249 (83%)	654 (17%)	
Previous stroke				
Yes	324	241 (74%)	83 (26%)	
No	5197	4278 (82%)	919 (18%)	
Operation type				
Aorta	198	167 (84%)	31 (16%)	
CABG	2117	1804 (85%)	313 (15%)	
CABG + other	1074	764 (71%)	310 (29%)	
Valve	1683	1421 (81%)	262 (16%)	
Valve + other	370	299 (81%)	71 (19%)	
Other	79	64 (81%)	15 (195)	
EuroSCORE 2	1.83 [1.78–1.87]	1.7 [1.0–3.1]	2.9 [1.6–6.3]	<0.001
CPB (min)	98 [97–99]	95 [75–122]	114 [88–151]	<0.001
AOX (min)	65 [64–66]	63 [49–84]	76 [55–105]	<0.001
ICU LOS (d)	1.2 [1.2–1.2]	1.2 [1.1–1.9]	3.3 [1.4–7.5]	<0.001
Hospital LOS (d)	9.11 [9.02–9.19]	8.3 [6.4–12.7]	13.1 [8.9–20.9]	<0.001

**Table 2:** Dosing cohort demographics. CPB = cardiopulmonary bypass, AOX = aortic cross clamp.

	A (*n* = 1779)	B (*n* = 2129)	C (*n* = 1613)	*p-*value
TXA (mg/kg)				
Cam −ve	19.4 (19.2 – 19.6)	24.4 (24.4 – 24.7)	29.9 (29.9 – 30.3)	
Cam +ve	19.4 (18.2 – 19.8)	24.7 (24.4 – 25.0)	30.3 (29.9 – 30.8)	
Age (years)				
Cam −ve	67 (67.0 – 68.0)	71 (71.0 – 72.0)	72 (71.0 – 73.0)	<0.001
Cam +ve	70 (69.0 – 71.0)	73 (73.0 – 74.9)	76 (74.0 – 77.0)	<0.001
Sex				
Male				
Cam −ve	1203 (67%)	1448 (68%)	670 (41%)	0.02
Cam +ve	242 (14%)	291 (14%)	161 (10%)	0.65
Female				
Cam −ve	260 (15%)	311 (15%)	629 (39%)	0.002
Cam +ve	75 (4%)	80 (3%)	153 (10%)	0.07
Left ventricular function				
Good	989 (55%)	1218 (58%)	892 (55%)	0.95
Cam −ve	171 (10%)	235 (11%)	186 (11%)	0.96
Cam +ve				
Moderate				
Cam −ve	406 (23%)	466 (22%)	327 (20%)	0.88
Cam +ve	108 (6%)	104 (5%)	106 (7%)	0.85
Severe				
Cam −ve	46(2.5%)	52 (2.5%)	57 (4%)	0.90
Cam +ve	26 (1.5%)	22 (1%)	14 (1%)	0.78
Very severe				
Cam −ve	21 (1.2%)	22 (1%)	23 (1.5%)	0.77
Cam +ve	12 (0.8%)	10 (0.5%)	8 (0.5%)	0.98
Hypertension				
Yes				
Cam −ve	823 (46%)	963 (45%)	638 (40%)	0.79
Cam +ve	195 (11%)	209 (10%)	171 (11%)	0.97
No				
Cam −ve	639 (36%)	795 (37%)	661 (40%)	0.89
Cam +ve	122 (7%)	162 (8%)	143 (9%)	0.88
Diabetes mellitus				
Yes				
Cam −ve	430 (24%)	382 (18%)	208 (13%)	0.19
Cam +ve	109 (6%)	105 (5%)	61 (4%)	0.82
No				
Cam −ve	1049 (59%)	1376 (65%)	1091 (67%)	0.76
Cam +ve	208 (11%)	266 (12%)	253 (16%)	0.58
Previous stroke				
Yes				
Cam −ve	79 (4%)	103 (4%)	65 (4%)	0.98
Cam +ve	23(1%)	36 (2%)	25 (1%)	0.78
No				
Cam −ve	1383 (78%)	1655 (78%)	1234 (77%)	0.98
Cam +ve	294 (17%)	335 (16%)	289 (18%)	0.94
EuroSCORE 2				
Cam −ve	1.5 (1.4–1.54)	1.6 (1.5–1.7)	2.1 (1.9–2.2)	0.55
Cam +ve	3.2 (2.7–3.8)	2.5 (2.3–2.8)	3.0 (2.8–3.5)	0.64
CPB (min)				
Cam −ve	98 (95.0–100.0)	96 (94.0–98.0)	92 (89.7–93.3)	0.91
Cam +ve	129 (121.6–134.4)	111 (107.0–116.0)	109 (102.1–113.9)	0.35
AOX (min)				
Cam −ve	65 (63.0–65.0)	63 (62.0–65.0)	61 (59.0–63.0)	0.94
Cam +ve	83 (75.3–88.0)	74 (69.0–79.0)	72 (68.0–76.0)	0.64

**Table 3:** Dosing cohorts length of stay (LOS).

	A (*n* = 1779)	B (*n* = 2129)	C (*n* = 1613)	*p-*value
ICU LOS				
Cam −ve	1.2 (1.2–1.2)	1.1 (1.1–1.2)	1.2 (1.2–1.2)	0.97
Cam +ve	4.3 (3.9–5.2)	3.1 (2.4–3.3)	3.1 (2.5–3.4)	0.25
Hospital LOS				
Cam −ve	8.4 (8.2–8.9)	8.2 (8.1–8.4)	8.4 (8.3–9.0)	0.98
Cam +ve	13.4 (12.1–15.1)	12.7 (11.3–13.6)	13.7 (12.2–15.2)	0.82

The association between cardiac surgery and PD is well established, as are several patient and procedure-related risk factors, such as age, history of pre-operative stroke, intra-operative red blood cell transfusion, or time spent on cardiopulmonary bypass (CPB) [3, 5, 6]. The mechanisms underlying the phenomenon of PD, however, are poorly understood [7]. Prevailing theories implicate micro-embolic events, altered perfusion, and pro-inflammatory responses associated with CPB and blood transfusion, as well as the surgical process itself, and having to undergo general anesthesia [8, 9].

Antifibrinolytic Tranexamic acid (TXA) is frequently used in cardiac surgery. It demonstrates efficacy in reducing bleeding and transfusion requirements and thus may reduce PD as a secondary effect [10].

In addition, there is emerging evidence that blood-brain barrier (BBB) dysfunction is not only seen regularly after cardiac surgery but also contributes to the development of PD [11–13]. Rudolph et al. found elevated chemokine levels in cardiac surgical patients developing PD [12], while Požgain et al. demonstrated that the neurotoxic accumulation of β-amyloid proteins can have an impact on the development of PD [14, 15].

TXA exhibits promise in ameliorating neuroinflammation and stabilising the blood-brain barrier through its inhibition of plasmin [16], which is known to promote brain inflammation [17]. The CRASH-3 trial found that TXA administered early not only reduces mortality in patients with mild-to-moderate head injuries but also improves surrogates of neurological recovery [16, 18]. The anti-inflammatory effect of TXA in trauma patients has been shown in several animal models as well as observational human studies [19]. This effect extends into elective hip and knee arthroplasty [20]. In adult cardiac surgical patients, TXA reduces multiple postoperative pro-inflammatory biomarkers [21], which in addition to stabilising the BBB might open new potential avenues for research into the prevention or at least decreasing the risk of PD after cardiac surgery.

## Materials and methods

This observational study was designed using the STROBE checklist.

### Study design and population

We conducted a single-centre retrospective cohort study involving adult patients (≥18 years) who underwent elective or semi-urgent cardiac surgery with cardiopulmonary bypass (CPB) at a quaternary cardiothoracic referral hospital between May 2018 and September 2022.

The inclusion criteria were broad, and only the following patients were excluded:

- Patients undergoing emergency procedures.
- Solid organ transplantation.
- Ventricular assist device implantation.
- Surgeries involving deep hypothermic circulatory arrest.
- Operations involving thoracic vessels other than the ascending aorta.

It is important to note that our study design did not include a control group without TXA administration. This is due to the National Institute for Health and Care Excellence (NICE) guidelines in the UK, which mandate the use of TXA in surgical procedures with a risk of moderate blood loss, including cardiac surgery [22]. As a result, all patients in our study received TXA, and we focused our analysis on the potential dose-dependent effects of the drug.

### Data collection

Anaesthesia and intensive care data were sourced from electronic medical records (Metavision, iMDSoft, Düsseldorf, Germany). Patient demographics, risk factors, surgical, and length of stay data were retrieved from an in-house database (CARDS II).

### Outcomes

The primary outcome was the association between the tranexamic acid (TXA) dose per kilogram of body weight and the incidence of PD within 48 h after surgery, assessed using the Confusion Assessment Method for the Intensive Care Unit (CAM-ICU) score [23]. CAM-ICU scores were routinely measured and documented at least four times daily as part of standard nursing observations.

ICU and hospital length-of-stay (LOS), as surrogates for morbidity, were collected as secondary outcomes.

### Anaesthesia and ICU protocol

None of the patients received any sedative pre-medication. Anaesthetic induction was achieved using a combination of benzodiazepine, opioid, and propofol. Maintenance was managed with a propofol infusion, and additional inhaled anaesthetics as directed by the consultant anaesthetist. A fixed 2 g bolus of TXA was administered before heparinisation for CPB, regardless of patient body weight.

Postoperatively, patients were weaned from CPB with the aid of epicardial pacing, inotropic, and vasoconstrictor therapy under the guidance of the consultant anaesthetist and surgeon. Heparin was reversed with protamine once circulation was re-established. Patients were then transferred to the ICU, where extubation was nurse-led based on predefined criteria.

### Perfusion technique

CPB was performed using LivaNova S5 machines with Sorin 8F oxygenators and phosphorylcholine-coated polyvinylchloride tubing (LivaNova, London, UK). The priming solution consisted of 2 L of Hartmann’s solution, 500 mL of 10% mannitol, and 5,000 units of heparin, reduced to 1 L post-priming to avoid unnecessary haemodilution. No additional TXA was added to the pump prime. CPB flow rates were maintained at 2.4 L/min/m^2^, with haematocrit levels kept above 22%. Procedures were conducted under normothermia or mild hypothermia (32–37 °C). Vasopressors were administered to maintain mean arterial blood pressure between 60 and 80 mmHg. Cardiac arrest was induced using blood cardioplegia with St. Thomas’ solution in a 4:1 blood-to-cardioplegia ratio.

### Statistical analysis

Continuous variables are presented as mean ± standard deviation (SD) or median and 95% Confidence Interval for parametric and non-parametric data, respectively. Comparisons within and between groups were conducted using Mann-Whitney and Kruskal-Wallis tests, while categorical variables were analysed using the Chi-square test.

Data were handled in MS Excel (Microsoft Corporation, Redmond, USA). Statistical analyses were performed using MedCalc software (MedCalc, Oostende, Belgium). The significance level was set at *p* < 0.05 for all tests.

## Results

7211 datasets of patients having cardiac surgery at Royal Papworth Hospital between 1st May 2018 and 30th September 2022 were extracted and analysed. After exclusion for duplicate entries or ineligibility, 5525 patients were included in our analysis (see Figure 1). The summary of patient demographics and operative data can be found in Table 1.

Overall, 4523 (82%) of all included patients had no evidence of confusion in the first 48 hours, while 1002 (18%) had at least one CAM-ICU positive entry in their electronic medical record within the first 48 h after cardiac surgery. The incidence of PD increases with the morbidity burden, as shown by the left ventricular function (LVF). While patients with good LVF have an incidence of PD in keeping with overall results, those with very poor LVF have almost double the incidence of PD (16% vs. 31%). This trend can also be seen in the operation type, where patients with isolated coronary artery bypass grafting (CABG) show a PD incidence of 15%, while those undergoing CABG plus another procedure have an incidence of 29%.

To investigate a dose-dependent effect of TXA administration before heparinisation on PD, we divided the 5525 patients included in the analysis into three groups, based on the mg/kg dose of the drug they received. Group A (*n* = 1780) received up to 22 mg/kg, Group B (*n* = 2130) 22.01–27 mg/kg, and Group C (*n* = 1615) 27.01 mg/kg or more. The demographic distribution between the three groups again follows the same pattern as that of the overall cohort (Table 2).

### Primary outcome

There was no statistically significant difference between the three groups in the incidence of at least one CAM-ICU positive entry in the patient’s EMR during the first 48 h. The percentage of CAM-ICU positive patients in each group was in keeping with the overall cohort (Group A = 18%, Group B = 17%, Group C = 20%, *p* = 0.25).

### Secondary outcome

There was no statistically significant difference between the three groups in ICU or hospital LOS in CAM-ICU positive or negative patients (see Table 3). However, being CAM-ICU positive was associated with prolonged ICU and hospital LOS across all three groups. None of the included patients experienced any seizures.

## Discussion

Earlier studies have indicated that TXA has both potential neurotoxic and neuroprotective effects [24]. Based on this previous work, this study aimed to investigate the relationship between the dose of TXA administered before CPB and the incidence of PD following cardiac surgery. We found no significant association between TXA dose and the incidence of PD, nor did we find a difference in the incidence of postoperative seizures between the three groups. There was no difference in the ICU or hospital LOS between the groups.

TXA is widely used in cardiac surgery. Its antifibrinolytic properties are associated with a reduction in bleeding transfusion requirements [10, 25, 26]. The introduction of TXA into widespread clinical practice was not without controversy. After the initial description of increased seizure activity in cardiac surgical patients exposed to high doses of TXA [24], numerous studies explored its utility beyond the principal haemostatic properties. Lecker et al. demonstrated TXA’s ability to influence neuronal excitability by inhibition of glycine and GABA receptors, proposing a mechanism by which TXA can potentiate the risk of convulsive seizures [27]. Lower doses have since been proposed and adopted, and more recent reports indicate no significant effect on seizure risk when TXA doses of up to 2 g are given [28, 29].

The evidence for using TXA to prevent PD after cardiac surgery is scarce and contradictory. In a propensity-matched study of 2757 pairs, a single 1 g bolus given during off-pump CABG did not show a difference in the incidence of PD when compared to a non-TXA group (4.2% vs. 5.0%) [30]. This is in stark contrast to a similar study involving 3392 on-pump CABG patients, which showed an increase in PD in the TXA group (9.9% vs. 7.1% [31].

More recently, additional properties of TXA, ranging from infection prevention to ameliorating neuroinflammation, have generated some excitement and have become the subject of academic interest [32, 33].

The proposed mechanisms for TXA’s potential neuroprotective effects and reduction in the development of PD include:

1. Inhibition of plasmin-mediated neuroinflammation: TXA, as a lysine analogue, inhibits the conversion of plasminogen to plasmin. Plasmin has been implicated in immune responses, neuronal function, and neuroinflammatory processes, and its inhibition may reduce the inflammatory cascade in the central nervous system [33–35].
2. Stabilization of the BBB: By reducing fibrinolysis and maintaining the integrity of fibrin clots, TXA may help stabilize the blood-brain barrier, potentially reducing the influx of inflammatory mediators into the central nervous system [16, 18, 34–37].
3. Modulation of excitatory neurotransmission: Some studies have suggested that TXA may interact with GABA and glycine receptors, potentially influencing neuronal excitability and susceptibility to excitotoxicity [27, 30, 36, 38, 39].

Despite these proposed mechanisms, our study did not find a dose-dependent protective effect of TXA against PD. One possible explanation for our findings is the multifactorial nature of PD in cardiac surgical patients [2]. While TXA may target specific pathways implicated in neuroinflammation and blood-brain barrier dysfunction, patient-related factors such as age, frailty, baseline cognitive status, and co-morbidities, as well as procedural factors such as CPB duration, perioperative haemodynamics, and inflammatory responses are difficult to influence. Indeed, the pathophysiology of PD is complex and likely involves a combination of ischaemic, inflammatory, and neurodegenerative processes [40–42].

In keeping with earlier studies, our study shows that patients who had at least one postoperative CAM-ICU positive episode were older and had longer CPB and aortic cross-clamping (AOX) times and a higher burden of co-morbidities. Despite an overall 18% of patients showing signs of PD, there was no difference in the incidence at three different, clinically accepted TXA dose ranges.

In our study, TXA was administered as a 2 g bolus before heparinisation for cardiopulmonary bypass, regardless of patient body weight. It is possible that alternative dosing regimens or timing of administration may yield different results. Reports in traumatic brain injury cohorts have shown that early administration of TXA leads to improved neurological outcomes [18, 36]. Future efforts could explore the optimal timing and duration of TXA administration to maximise its potential neuroprotective effects.

### Limitations

As a retrospective cohort study, our findings are subject to inherent biases and confounding variables. The reliance on electronic medical records for data collection may have introduced inaccuracies or missing information.

The National Institute for Health and Care Excellence (NICE) mandates the use of TXA in surgical procedures where there is a risk of moderate blood loss, including cardiac surgery [22]. In addition to the retrospective design, the absence of an untreated control group is a significant limitation of our study. While this NICE guidance ensures that all patients receive standard-of-care treatment, it restricts our ability to compare cognitive outcomes between TXA-treated and untreated patients in a prospective, randomised, controlled manner. Future studies may need to consider alternative study designs or international collaborations to address this limitation. The multicentre randomised controlled TRIGS-D trial may provide valuable insights into the effects of TXA on PD, as it evaluates whether the prophylactic administration of TXA in patients undergoing major gastrointestinal surgery reduces the incidence. Crucially, this study compares TXA administration against placebo, addressing the significant limitation of our study [43].

PD after cardiac surgery is multifactorial, and some academic effort has gone into identifying risk factors. This is a retrospective database study and can necessarily only incorporate data that has been collected. The authors acknowledge that important factors like patient hemodynamics or transfusion have not been assessed. The design of a prospective study needs to consider these additional factors.

Another limitation is the generalisability of our findings. Although our patient cohort was diverse, the single-centre nature of the study may limit the applicability of the results to other settings.

Recent research has highlighted a number of non-haemostatic characteristics of TXA, focusing on its ability to reduce neuroinflammation and, with it, potentially the incidence of PD. The present study does not support the theory that doses in the higher clinically acceptable range decrease the incidence of PD after cardiac surgery on CPB. It is, however, limited in its relevance by the retrospective design without an untreated control group.

Future research, such as the TRIGS-D trial, where ethically and practically feasible, will be crucial in establishing the impact of TXA on postoperative delirium.

## Data Availability

Study data is held securely at Royal Papworth Hospital and is available in anonymised form upon request.
